# Preliminary findings on the experiences of care for parents who suffered perinatal bereavement during the COVID-19 pandemic

**DOI:** 10.1186/s12884-021-04292-5

**Published:** 2021-12-22

**Authors:** Sergio A. Silverio, Abigail Easter, Claire Storey, Davor Jurković, Jane Sandall, on behalf of the PUDDLES Global Collaboration

**Affiliations:** 1 Department of Women & Children’s Health, School of Life Course & Population Sciences, Faculty of Life Sciences & Medicine, King’s College London, London, UK; 2 International Stillbirth Alliance, Bristol, UK; 3 Gynaecology Diagnostic Outpatient Treatment Unit, University College Hospital, University College London Hospitals NHS Foundation Trust, London, UK

**Keywords:** Pregnancy loss, Perinatal death, Miscarriage, Stillbirth, Neonatal death, COVID-19, Maternity care, Service reconfiguration

## Abstract

**Background:**

The COVID-19 pandemic poses an unprecedented risk to the global population. Maternity care in the UK was subject to many iterations of guidance on how best to reconfigure services to keep women, their families and babies, and healthcare professionals safe. Parents who experience a pregnancy loss or perinatal death require particular care and support. PUDDLES is an international collaboration investigating the experiences of recently bereaved parents who suffered a late miscarriage, stillbirth, or neonatal death during the global COVID-19 pandemic, in seven countries. In this study, we aim to present early findings from qualitative work undertaken with recently bereaved parents in the United Kingdom about how access to healthcare and support services was negotiated during the pandemic.

**Methods:**

In-depth semi-structured interviews were undertaken with parents (*N* = 24) who had suffered a late miscarriage (*n* = 5; all mothers), stillbirth (*n* = 16; 13 mothers, 1 father, 1 joint interview involving both parents), or neonatal death (*n* = 3; all mothers). Data were analysed using a template analysis with the aim of investigating bereaved parents’ access to services, care, and networks of support, during the pandemic after their bereavement.

**Results:**

All parents had experience of utilising reconfigured maternity and/or neonatal, and bereavement care services during the pandemic. The themes utilised in the template analysis were: 1) The Shock & Confusion Associated with Necessary Restrictions to Daily Life; 2) Fragmented Care and Far Away Families; 3) Keeping Safe by Staying Away; and 4) Impersonal Care and Support Through a Screen. Results suggest access to maternity, neonatal, and bereavement care services were all significantly reduced, and parents’ experiences were notably affected by service reconfigurations.

**Conclusions:**

Our findings, whilst preliminary, are important to document now, to help inform care and service provision as the pandemic continues and to provide learning for ongoing and future health system shocks. We draw conclusions on how to enable development of safe and appropriate services during this pandemic and any future health crises, to best support parents who experience a pregnancy loss or whose babies die.

**Supplementary Information:**

The online version contains supplementary material available at 10.1186/s12884-021-04292-5.

## Background

Pregnancy presents a unique time across the lifecourse. Though usually an occasion of great joy, pregnancies can be physically and emotionally turbulent when women experience complications [1], or go onto suffer a miscarriage, stillbirth, or neonatal death, the effects of which are often devastating. The global Coronavirus [SARS-CoV-2] pandemic (COVID-19) poses an unprecedented risk to health and significant disruption to healthcare, including maternity, neonatal, and bereavement care services. The final cost of the global pandemic to human life is yet to be totalled and it may take some time before there is a more nuanced understanding of the true psychological impact of those who survive the pandemic. For perinatal women, COVID-19 has caused *“unprecedented challenges that can significantly impact on women’s mental health”* ([2]; p.310). Given the negative impact of both pregnancy loss and perinatal death [3, 4], and the pandemic on maternity care provision [5, 6], investigation of how bereaved parents have experienced reconfigured services is crucial to future learning, providing important insights for service provision.

### Pregnancy loss and perinatal death

In the UK, approximately 750,000 live births occur every year [7], with 12-24% of all clinically recognised pregnancies ending in miscarriage [8], 1% of all pregnancies resulting in an ectopic [9], and approximately 2500-2800 stillbirths and 1100-1250 neonatal deaths reported each year [7]. Collectively, stillbirths and neonatal deaths are often referred to as ‘perinatal deaths’, as opposed to miscarriages which are broadly categorised as a ‘pregnancy loss’. Definitions of pregnancy losses (miscarriages) and perinatal deaths (stillbirths and neonatal deaths) are subject to global variation [10], which adds complexity when attempting to compare experiences internationally. For example, the World Health Organization [11] defines a stillbirth as a baby born with no signs of life ≥28^+ 0^ weeks’ gestation, but Australia recognises stillbirths from 20^+ 0^ weeks, whereas some countries (e.g. Italy) do not have a specific terminology for ‘stillbirth’, instead using the broader ‘intrauterine deaths’. Meanwhile, in the UK, pregnancy losses of up to 23^+ 6^ weeks are classified as ‘miscarriages’, and do not entitle parents to maternity or paternity leave [12], nor may the parents formally register their baby [13]. Regardless of definition, the experience of pregnancy loss and perinatal death can be devastating, with wide-ranging and frequently long-lasting effects on the psychological wellbeing of parents and on those around them [1, 14–19], with the psycho-social implications of stillbirth recently being discussed as *“overlooked and underappreciated”* ([4]; p.604) and the psychological consequences of miscarriage being reported as going *“unrecognised by health-care professionals, family, and friends”* ([3]; p.1663).

### The global COVID-19 pandemic

To reduce transmission of the COVID-19 virus and preserve population health, countries across the globe implemented various public health strategies. These included social and physical distancing; mask-wearing; stay-at-home orders (‘lockdowns’); furlough schemes; quarantine procedures; and ‘shielding’ (stay-at-home recommendation for extremely clinically vulnerable). Initially, the recommendation to ‘shield’ was extended to pregnant and postpartum women (that is to say, all pregnant women were advised to stay-at-home under all circumstances except when accessing emergency medical care or giving birth) [20]. In the UK, this guidance was revoked when evidence emerged suggesting, in the absence of clinical vulnerability, pregnant women were no more susceptible to the virus than the general population [21]. However, it has been affirmed women in their third trimester or those pregnant women from Black, Asian, or Minority Ethnic [BAME] backgrounds are susceptible to a more severe illness with greater symptomatology, should they contract the virus [21, 22]. Current guidance now stipulates pregnant women in their third trimester (≥28^+ 0^ weeks of gestation) or with underlying health conditions, should continue to minimise contact with others despite the removal of social distancing restrictions in the UK [22].

The health system shock as caused by the COVID-19 pandemic has caused significant disruption to maternity care around the world. In the UK, these changes have been consistently monitored [23] and guidance as well as service provision has been frequently adapted in an attempt to augment care [5, 21–23]. For perinatal women receiving maternity care, we have already seen reports of devalued care [5], poor psycho-social outcomes [6, 24], and worrying projections suggesting rates of stillbirth are expected to double globally [25].

### The present study

**Fig. 1:**
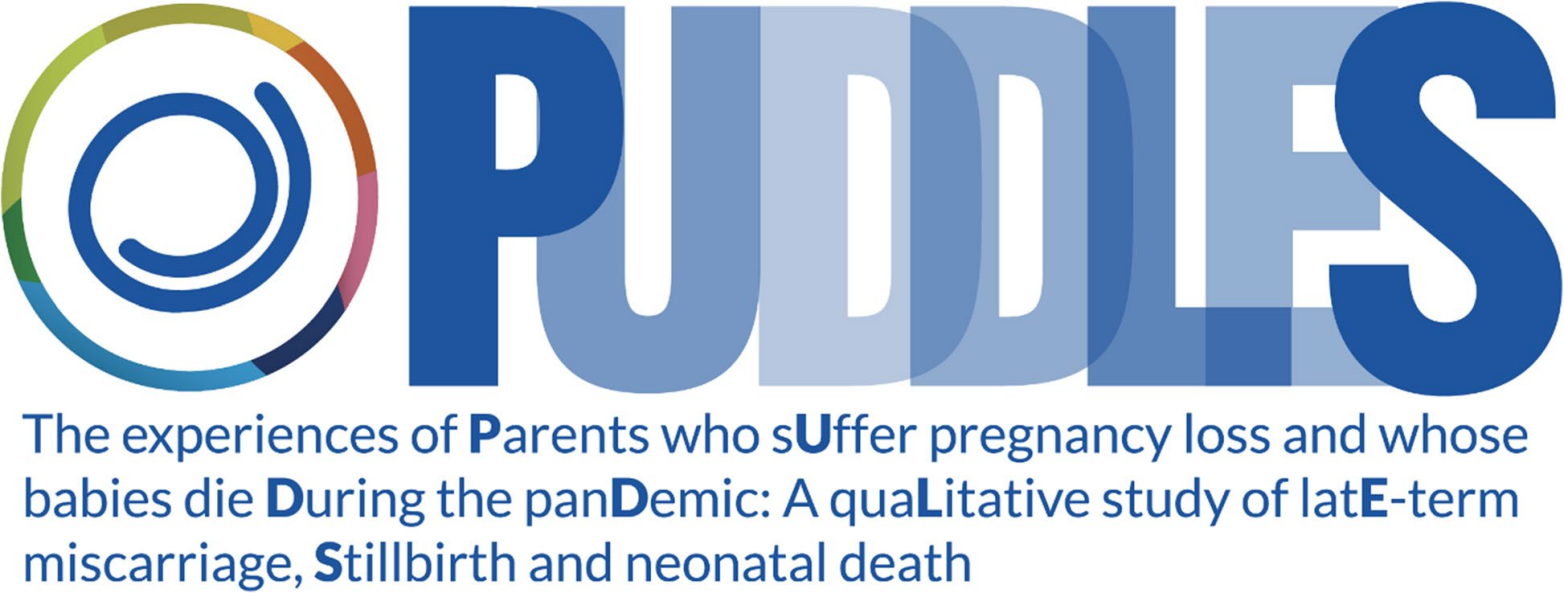
PUDDLES Global Collaboration logo

To undertake such investigations on an international level, The Centre of Research Excellence in Stillbirth [Stillbirth CRE] in Australia initiated a collaboration called *‘Continuing care in COVID-19 Outbreak: A global survey of new and expectant parent experiences’* [COCOON]. COCOON was devised as a large, multi-country, on-line survey for pregnant and postpartum women and their partners, and parents who experienced a stillbirth or neonatal death, from 30 January 2020 onward (the results of which will be reported elsewhere). As part of this collaborative work, King’s College London devised and has led on a nested qualitative interview study called *‘The experiences of Parents who suffer pregnancy loss and whose babies die during the pandemic: A qualitative study of late-term miscarriage, stillbirth, and neonatal death’* (PUDDLES; see Fig. 1).

**Fig. 2:**
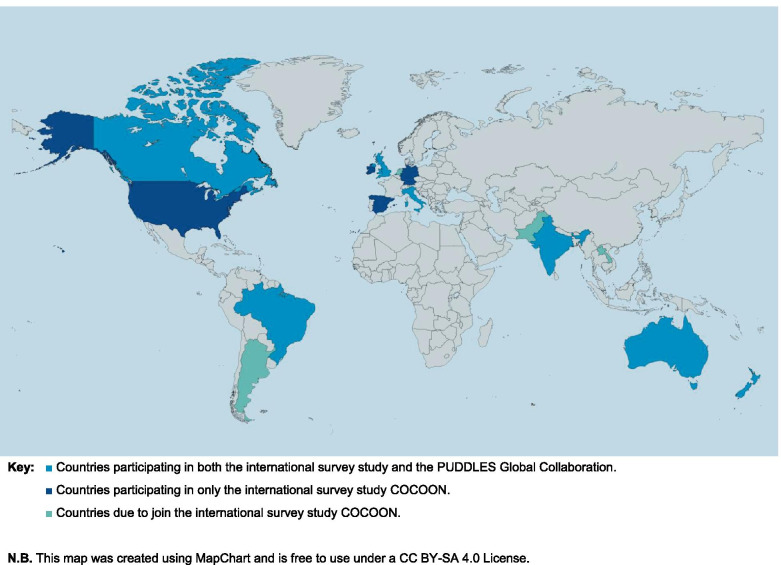
Countries involved in the PUDDLES Global Collaboration

The on-line survey format of COCOON allowed for minor country-to-country variations, including the option to recruit participants to take part in PUDDLES. The PUDDLES Global Collaboration, comprises self-selected psychologists, midwives, obstetricians, nurses, social scientists, public health specialists, health service and system researchers, and bereaved parents from seven countries who are participating in the larger COCOON survey study (United Kingdom, Australia, Brazil, Canada, India, Italy, & New Zealand; see Fig. 2).

In this paper, we report on the first data collected as part of the PUDDLES Global Collaboration. In doing so, we present an analysis of interview data from the UK, reflecting bereaved parents’ experiences of accessing services, networks of support, and care.

## Methods

In this study, we utilised broad categories from an international mapping exercise (the results of which will be reported elsewhere) as a template for analysis of interview data from the first wave of the PUDDLES-UK interview study (November-December 2020). Ethics approval for PUDDLES-UK were sought and granted from the King’s College London Biomedical & Health Sciences, Dentistry, Medicine and Natural & Mathematical Sciences Research Ethics Sub-Committee [ref:- HR-19/20-19455].

### Patient and public involvement and engagement

This programme of work has been presented to, and received input from the National Institute for Health Research Applied Research Collaboration [NIHR ARC] South London Patient and Public Involvement and Engagement [PPIE] meeting for Maternity and Perinatal Mental Health Research (July 2020; June 2021), which has a focus on co-morbidities, inequalities, and maternal ethnicity. Further input has been received from the Chief Midwifery Officer and the Maternity Transformation Team of NHS England and NHS Improvement at a meeting focused on multi-morbidities and maternity safety (July 2020) and from an NIHR ARC South London Work in Progress Meeting (October 2020) focusing on maternity and perinatal mental health research. Throughout the course of planning and undertaking this work we have received feedback from both lay and expert stakeholders, including members of the public, those with lived experience, health and social care professionals, researchers, and policy makers.

### Recruitment and participants

For countries participating in the on-line survey study, who also opted to participate in the PUDDLES Global Collaboration qualitative interview study, there was a page at the end of the survey for bereaved parents to leave contact details to indicate willingness to take part in follow-up interviews. Parents in the UK who had a late miscarriage (14^+ 0^-23^+ 6^ weeks’ gestation) were not included in the on-line survey, but could participate in interviews by leaving contact details once screened-out of the survey. Participants subsequently provided informed consent for PUDDLES (electronically signed or audio recorded). Participants (*N* = 24) had either experienced a late miscarriage (*n* = 5; all mothers), a stillbirth (*n* = 16; 13 mothers, 1 father, 1 joint interview involving both parents), a or neonatal death (*n* = 3; all mothers). Participants were de-identified, and demographic data (e.g., age, educational attainment, ethnicity, geographical location, religion, etc.) were deliberately not recorded nor presented in order to preserve confidentiality of participants. This decision was taken due to there being relatively few stillbirths and neonatal deaths in the UK each year (fewer than 4000 combined) [7], and reporting and/or linking demographic data to participants in a qualitative study such as this, with a relatively large sample size, has the potential to provide an opportunity for identifiability. We therefore reassured participants that demographic data would not be requested, nor reported upon if volunteered spontaneously.

### Data collection

Interview schedules were developed by the lead author [SAS] who has experience of undertaking interviews on sensitive topics (including pregnancy loss), in discussion with the wider team [CS, JS, AE], as well as members of the PUDDLES Global Collaboration and our charitable partners (Tommy’s, Sands, International Stillbirth Alliance). The schedules were internally sense-checked by our charitable partners and collaborative researchers rather than piloted, to ensure participants’ data were not lost to pilot interviews, given our initial expectation of a small recruitment population.

Interviews were conducted between November and December 2020, via video-call or telephone by the lead author [SAS] who is an experienced, male researcher with responsibility for the provision of qualitative research training and improvement across their academic School and its five research Departments. Interviews were recorded, and audio verbatim-transcribed (*M*_*Time*_ = 72 min), with memo notes being taken by hand and added to the bottom of transcripts after transcription. Interview schedules (see Additional files 1, 2, and 3) had similar questions, but differed according to the context of the type of bereavement (Miscarriage – Additional file 1; Stillbirth – Additional file 2; Neonatal Death – Additional file 3). Interviews covered parents’ experiences of pregnancy loss or perinatal death, access to services, and support; and were semi-structured, to allow flexibility whilst ensuring common lines of inquiry [26].

### Data analysis

Interview data from UK-based bereaved parents were analysed [SAS] using a template analysis [27–29], based on a mapping exercise which documented the national responses to COVID-19 and service reconfigurations in maternity, neonatal and bereavement care, as issued by respective national Governments. All parents had suffered a late miscarriage, stillbirth, or neonatal death during the COVID-19 pandemic. Preliminary coding was checked with the wider team [CS, JS, AE, DJ] before higher-order coding of themes was shared with the rest of the PUDDLES Global Collaboration. Researcher bias was reduced by a process of ‘bracketing’ [30], whereby personal assumptions were recorded and excluded as far as possible, with all members of the PUDDLES Global Collaboration iteratively checking the findings and results, whilst highlighting any sources or examples of potential bias for removal.

**Fig. 3:**
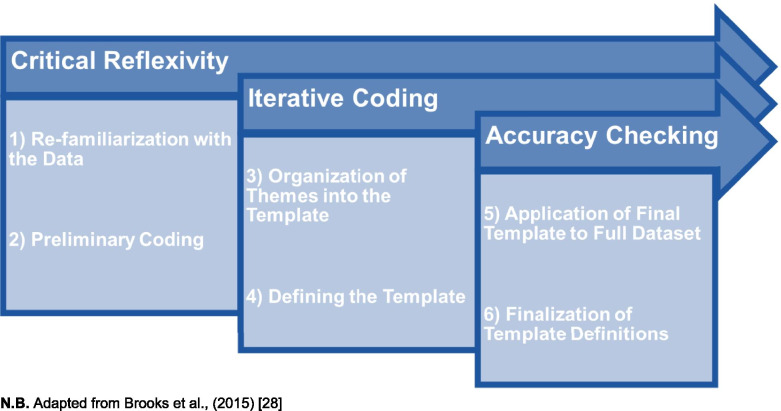
The Template Analysis Methodology

Template analysis as a method of qualitative data analysis is philosophically flexible, but has been employed in this study using a critical perspective ([29]; p.15), whereby we argue *“realities which exist independent of human activity”* and that situations outside of our participants’ control (such as the COVID-19 pandemic in the case of our study) *“may not directly determine behaviour, they are nonetheless recognized as having important influences in understanding experience”*. Template analysis follows a six-stage process: 1) Re-familiarization with the Data; 2) Preliminary Coding; 3) Organization of Themes in the Template; 4) Defining the Template; 5) Application of the Final Template to the Full Dataset; and 6) Finalization of Template Definitions; thus making template analysis a highly methodical approach to analysis. Template analysis maintains rigour by ensuring critical reflexivity is engaged with throughout all six phases; as well as iterative coding is practiced from stage three onwards; and ensuring accuracy checking in the final two stages ([28]; see Fig. 3).

Interview transcripts from UK-based bereaved parents were therefore electronically ‘hand-coded’ using template analysis [27] on Microsoft word, utilising key points of interest in the mapping exercise: national responses to COVID-19; and maternity, neonatal, and bereavement care reconfigurations. All transcripts were re-read as part of the re-familiarization process. The initial template was developed based on the broad themes found in the knowledge mapping exercise, and were organised into a coding template. They were then given definitions which could be overlaid onto the original key areas of interest found in the knowledge mapping exercise. This template was then applied across the whole interview dataset before definitions the template being finalized with the addition of psycho-social descriptors which were more reflective of the data, applied to the template as theme names.

Saturation of data was assessed on two axes: data saturation, whereby similar data was present across many, if not all of the participants relatively early on into recruitment; and thematic saturation, whereby the final themes were well supported by data contained within the whole dataset. Although this analysis is reported as preliminary, we were satisfied that both parameters were met and that themes were broadly applicable across the whole dataset. The iterative approach to coding and quotation selection meant, when saturation of data and themes had been reached, transcripts could be compared to ensure the most illustrative quotations were selected and presented in the results section which follows [29].

## Results

We present only preliminary findings from the first wave of PUDDLES-UK qualitative interview data. These preliminary qualitative findings from the UK in the context of global maternity, neonatal, and bereavement care reconfigurations provide a rapid appraisal of the situation to aid and inform the ongoing development of safe and appropriate services as the pandemic continues.

### The shock & confusion associated with necessary restrictions to daily life

The national response to the COVID-19 pandemic was surprising to many, almost immediately halting life as they knew it. Many restrictions were placed on daily life meaning pregnant and women and their partners often sudden found themselves at home having to juggle the pregnancy, work, other caring responsibilities, and eventually their bereavement, all from their household with no ability to leave their homes. Many participants discussed how the first UK lockdown came with confusing messaging as to whether pregnant women were or were not at greater clinical risk of COVID-19:

*“In March when the lockdown happened and very abruptly pregnant women were put on the vulnerable list, that is when I began to become a bit more concerned about what was happening. I immediately had to leave work and go and work from home prior to the rest of my team following suit. Leaving abruptly in a bit of a panic, not realising at the time that I would never go back into that office, so I don’t know that I brought the appropriate things home. It all was radical and abrupt.”*

(P-001:Mother-Stillbirth)

*“They wanted me to take my maternity leave early. I said, ‘Well, I’m not taking my Mat leave early because I don't feel like I should have to. It’s not my fault this has happened.’ So basically, I took unpaid leave instead for four weeks, and then my Mat leave started……… During the lockdown I think I just got progressively more… It’s quite a weird thing to isolate in your house. I think because I was pregnant, I started getting more and more neurotic about [laughs] what people were touching and what was going on. Yes, so I think that got worse and worse. So, I just had the two appointments. We have a big garden, so I just would go out to the garden, but the only time I left the house was to go to the midwife appointment. It just got a bit mental, now looking back, but at the time I thought I was doing the right thing as far as protecting my baby. We were getting all of our shopping delivered, and instead of resting I was washing all the shopping down and stuff [laughs].”*

(P-007:Mother-Stillbirth)

However, whilst the new restrictions meant some felt trapped or dissatisfied, others used the restrictions as an excuse to not socialise when they did not want to, either during their pregnancy, or in their time of grief:

*“We used the pandemic in some senses as a bit of a safety net as well because we could use that as an excuse. ‘Oh, well, we can’t come out because we’re in lockdown’, or, ‘we’re isolating.’”*

(P-008:Mother-Stillbirth)

Navigating grief in the midst of the lockdown restrictions on social gatherings was coupled with the need to seek support, and occasionally bereaved parents were advised to stretch the rules to ensure they could still access support networks:

*“One thing that does stand out, we were told by our midwife to… I won’t say ignore lockdown, but she said, ‘Go out when you need to. See people when you need to. Look after your mental health in a time like this.’”*

(P-015:Father-Stillbirth)

*“My husband’s family live in… Well his sister lives in <City> so she could come and see him, no problem, but his parents live in <Town> and they called the police to get permission to come, because obviously you were not allowed to drive more than five miles. And the police said, ‘We can’t give you permission to do that. But if you do it and we stop you; we are probably not going to fine you.’ So, they could not even get permission to come. But they did anyway, as you would. So, they came, and they saw him for ten, fifteen minutes. And my husband’s sister met him for half an hour or so. But my parents live in other countries and they couldn’t come in time…”*

(P-006:Mother-Neonatal Death)

However, this sometimes caused conflict between bereaved parents and other people:

*“…we had actually had a neighbour report us for being at my mum and dad’s house and the police turned up… This is four days after we had got home from the hospital. So, the neighbour, we could see her peering over the hedge, and she had rung to say ‘they’ve got their daughter and son-in-law and the kids with them’……… she thought we were having a gathering. So, the police obviously came, and they were lovely. Obviously, given the circumstances my mum let them know.”*

(P-018:Mother-Stillbirth)

### Fragmented care and far away families

Maternity care changed rapidly with COVID-19 lockdown restrictions, leaving bereaved parents alone, isolated, and/or apart from their familial or fictive kin networks they would have relied on outside of pandemic circumstances. Often these service configurations in maternity care led to fragmented or cancelled care:

*“I think it was the hospital weren't completely able to give me what they had said that they would……… I never actually saw my consultant until I was 20 weeks. So, whether that was due to the pandemic, but every time I went up to the hospital for my antenatal appointments it was always a different midwife and a different consultant that saw me. I never got seen twice by the same person. I would have preferred to have continuity of care. They did allude to me, that was due to the pandemic because all the working shift patterns got changed quite quickly…”*

(P013:Mother-Late Miscarriage)

*“The most significant thing that happened in terms of my care was that several of my appointments were very immediately cancelled. Anything that was deemed routine and not medically necessary was cancelled. Then I believe two of my appointments from 30 weeks or maybe 26 weeks onwards were conducted over the phone. I was deeply concerned about this because I thought: ‘How on earth can you check anything over the phone? Aren’t you meant to be checking my urine, my blood, my blood pressure? How can you tell if something is not right?’”*

(P-001:Mother-Stillbirth)

For many months, partners were not allowed to be present at antenatal care visits and scans, even when parents were concerned about their baby:

*“Because we’d had the reduced movement again that day, well we thought that’s what it was, I was outside in the car park when my wife went in for what we thought would be a check and a scan, because in a strange way I suppose we’d kind of grown a bit, I don't know, used to him having these bouts of reduced movement. I didn’t really know much about how serious it was because he’d always been fine afterwards……… I remember I had my daughter in the car with me, but also because of lockdown and him being overdue we had my brother who lived nearby who was kind of on stand-by around the clock for us……… So, the hospital rang me and said that they needed me to come in. At which point I rang my brother and said I was dropping my daughter off.”*

(P-015:Father-Stillbirth)

When parents were told their baby had died, some healthcare professionals returned to tactile comforting, breaking COVID-19 protocols on physical distancing:

*“So, after that, I was moved from the scanning room into a room pretty much by myself. The registrars were called, and a consultant was called. They came in and met me and said their condolences as well. And from that point, we waited for <Husband’s Name> to get to the hospital. We talked a little bit about options, what could happen, and we decided to move forward with being induced a few days later. And then from there, the bereavement midwife came in. We had a fabulous bereavement midwife……… She provided us information from Sands. She was probably not risk-averse. She gave me a massive hug [laughs] with all her PPE on and everything else and told me she was so sorry, and really from that point on I felt like I started to bond with <Midwife’s Name>, because she was pretty much our point of contact with anything that was going on with the hospital.”*

(P-012:Mother-Stillbirth)

Whilst fathers and partners were usually able to stay during labour, some parents experienced difficulty negotiating the presence of their preferred birthing partners, which added to the distress of starting labour knowing their baby had died:

*“Yes, there weren’t any partner restrictions at that point, so I managed to stay for the entire labour, the entire delivery. I don’t think we had any restrictions, did we, at all at that stage?” (P-017) “Well, just that no one else was allowed, but I don’t know how I would have coped with <Husband’s Name> not being able to be there at any stage of that really.” (P-016)*

(P-017:Father & P-016:Mother-Stillbirth)

*“My sister, they wanted her to go at one point and I was like, ‘No, she is not going. You can’t do that.’ They were like, ‘No, because of Covid…’ Then I kicked up a fuss and my older sister spoke… I think she popped downstairs, and they said she can’t come. She spoke to the ward manager and because of my situation they allowed her to stay…”*

(P-003:Mother-Stillbirth)

### Keeping safe by staying away

Parents whose babies were ill and subsequently died also experienced neonatal service reconfigurations, whereby reduced visitation to a baby in neonatal care was often seen as a way of reducing the risk of COVID-19 infection. These restrictions were varied in nature, but were often imposed quickly and without advance warning:

*“When we were in NICU, we found it really valuable talking to the other parents. Then when the visiting restrictions and everything became much more tightly… when we weren't then allowed to use the parents’ room in NICU and there wasn’t any standing in the corridor and chatting to other parents, you’d got masks on, you’d got aprons and gloves and things, suddenly we then lost a support network that we had kind of started to rely on.”*

(P-011:Mother-Neonatal Death)

When babies were transferred between hospitals, parents had to navigate travel and testing for COVID-19 between the hospitals:

*“Yes. It is a weird, different time zone almost that hospitals work in. In the real world, six hours is nothing, but when it is quite literally life or death situations it feels like forever. They were waiting back to hear about things and trying not to give us any false hope, just giving us the facts. I was aware I was being managed, but I didn’t feel that was inappropriate. They didn’t have the answers……… They were working within difficult constraints as well with the virus and moving between one hospital and another there was a possibility that we had COVID. There were all the tests that we had to have on arrival……… They were having difficulty speeding those tests through so we could all be together. They did the best they could in really challenging circumstances.”*

(P-024:Mother-Neonatal Death)

Lockdown restrictions meant contacts were quickly minimised, meaning no-one except parents were allowed to attend babies in NICU:

*“For the first 30 days of his life, my parents had been to visit him every day in NICU. So then when visiting had been restricted, that had a big impact for them not being able to see him.”*

(P-011:Mother-Neonatal Death)

Restrictions on neonatal services also meant parents would often need to take shifts to be with their baby. This often led to confusion and mixed-messages, where one parent would have to ‘translate’ any updates to the other as they swapped over:

*“And then with the NICU we were not allowed to see him together. So, it was one parent at a time at the bedside, which sounds fair if you do not think about it very carefully, but when you are in a room with six other babies and they are doing a ward round and you are trying to remember these really complicated technical things that are happening to your baby, like how much they weigh, what antibiotics they are getting, how much food they are getting… Only one of us could be there at any one time so then we would have to go out and explain to the other one what the doctors had said during a ward round. And we are not doctors, we are not technical people, so having to explain that, things get lost in translation.”*

(P-006:Mother-Neonatal Death)

### Impersonal care and support through a screen

Safety precautions including personal protective equipment meant interactions between bereaved parents and healthcare professionals felt impersonal, rendering bereavement care less personable and in some cases less than adequate. Occasionally parents were distressed when unable to identify who was tending to their baby:

*“But one thing that I found made it harder was that you couldn’t see anybody’s face because obviously they were wearing masks. It makes everything a lot more impersonal. You know, if you are only seeing this part of somebody’s face you can’t… I don’t know, sometimes I was getting confused between who was who and I was thinking ‘Have I seen you before?’ I couldn’t really tell……… when the other nurse came in with the mask on and said that she would have to take <Baby’s Name> down, because <Midwife’s Name> was finishing her shift, I just got really upset. And then I did actually ask her, ‘Can I see your face?’… She said, ‘Yes of course you can.’ So, she pulled her mask down.”*

(P-014:Mother-Late Miscarriage)

Grieving alone was also discussed as distressing, especially when support was restricted:

*“So, the matron did allow his grandparents to come and meet him. They only were allowed half an hour to see him, so it was timed which obviously was horrendous, to have to say, ‘You have to leave now.’ The matron was very strict on that, so she did watch the clock……… It did change a lot of… I feel there was a barrier because obviously everyone was wearing masks, everyone was wearing visors. You couldn’t really have the relationship with the midwives. My midwife, who was there for the delivery, was really good. She delivered the care as best she could……… But I do feel like that was a big thing for me. I felt like it was very clinical. Which, I know it’s in a hospital, but I do feel like that was a big factor in our care, that it didn’t feel personal. It felt like we were just a number. It didn’t feel like <Baby’s Name> was our son; he was just another baby that had died, type of scenario. That is how it felt, and it all felt very… They were watching the clock all the time. They kept saying, ‘We don’t know if we can do that because of the guidelines. We don’t know where we stand with things.’ So, a lot of it was impacted… because of COVID.”*

(P-018:Mother-Stillbirth)

And it was only when lockdown restrictions eased, and limited travel and social gatherings were once again permitted, that parents reported feeling they could honour their baby’s passing in a way they had hoped:

*“We scattered his ashes, then thankfully lockdown had eased enough for us to go and do that. So generally, as I said, we unfortunately had been used to heartbreak and disappointment. We hadn't quite appreciated that it could happen so late so that was a whole new level of pain.”*

(P-005:Mother-late Miscarriage)

*“They [colleagues] have been very sensitive to my feelings, to what’s going on. They have sent me massive bouquets of flowers, when they first heard that <Baby’s Name> had passed away. Three of the team came to [his] funeral. My apprentice wrote a wonderful poem about <Baby’s Name> and stood up and read it at the funeral [sobs]. They came back to my garden for a bit of a gathering afterwards. It was lockdown, so we had a very socially-distanced brief gathering, so that was nice.”*

(P-011:Mother-Neonatal Death)

Bereaved parents often reported reaching out to external professional support, delivered virtually. Likewise, family interactions were usually conducted virtually, meaning all grief was navigated through a screen or via telephone:

*“About four weeks after our loss when I realised that we were probably in a bit of a mental health crisis, I found the charity Petals on-line and I contacted them and within a couple of days they had got back to me. And within I think even a week we were booked in for our first counselling session. That has been so crucial to us feeling human again really……… our friends and family have been hugely supportive, but I think for our family especially they were grieving too. They felt quite helpless because they couldn’t be here with us because of lockdown and they didn’t really know how they could support us at that stage………”* (P-016) *“I think when we felt up to seeing people, the fact that we couldn’t see people didn’t help. But I don’t think it necessarily…”* (P-017) *“And it took some of the pressure off when you didn’t feel like being particularly social because it didn’t feel like you needed to be because you couldn’t. But like <Husband’s Name> says, just being able to spend a bit of time with family or friends when you did feel up to it and not being able to, was maybe a bit frustrating at times. We did lots of Zooming [laughs].”* (P-016)

(P-016:Mother & P-017:Father-Stillbirth)

Video-conferencing was also suggested by hospitals to deliver post-mortem results which were frequently subject to delay due to the pandemic:

*“...they did say it [post-mortem] could take longer because of the pandemic……… They basically only invited me in, and I said, ‘I really want my husband to come’……… I said, ‘Well if he can’t come then I would rather have it via Zoom so that he can be with me, but my preference is we are face-to-face, and my husband is there’. They said, ‘Yes, given the circumstances, that’s fine’……… we were all masked up and you kind of can’t read… It wasn’t that comfortable, but it was necessary, I guess, because there was no mass testing… So, it was face-to-face, and he was allowed to come, which I am grateful for.”*

(P-025:Mother-Stillbirth)

## Discussion

To our knowledge, this is the only international collaboration to focus on documenting the experiences of bereaved parents after late miscarriage, stillbirth, or neonatal death, during the pandemic. We present rapid, but methodically analysed interview data from the UK, which is the first country within the PUDDLES Global Collaboration to have collected qualitative data from parents who suffered a late miscarriage, stillbirth, or neonatal death during the pandemic and the respective lockdowns over 2020. Our findings are explained, and conclusions drawn only in relation to (limited) published data on parental bereavement and service reconfiguration during the pandemic to date.

Firstly, our analyses elucidate the necessary restrictions to daily life imposed by governments to contain the spread of COVID-19. Qualitative data from the UK in this study, described how changes to daily life arrived quickly, sometimes with little-to-no time to adjust. Many participants suddenly began working from home or stopped working altogether. Restrictions on movement were double-edged for many; lockdowns providing the conditions and excuse to grieve privately, but also restricted family gatherings and access to usual support networks. These restrictions were similarly felt by many parents receiving maternity care, both globally [31, 32] and also in the UK [5, 6]. Similarities can also be drawn with other work in the study of bereavement, where the pandemic has redrawn social geographies and grounded people in their homes [33]. With the advent of home working and video-call socialising, the landscape of the home has changed, and therefore no longer stands as a safe and private space where parents could grieve [34], rather making them feel isolated [35].

Analyses relating to maternity care reconfiguration, extended the idea of the pandemic causing isolation and loneliness with fragmented care and faraway families. As in other research about maternity care during the pandemic [5, 6], women frequently reported the detrimental effects of increased virtual care and decreased face-to-face care. These changes were often discussed in interviews as leading women to feel alone during their pregnancy, especially when they found out their baby had died, whilst their partners had been confined to the carpark. Many parents in this study commented on these types of separation whereby partners had to remain in cars whilst women sought care, which has similarly been reported in other such maternity care studies [5]. Restrictions such as these, made women fearful they might have to deliver their miscarried or stillborn baby alone. Such fears echo previous findings from pregnant populations in Italy [36] and the USA [37].

Similar limits and restrictions were commonplace in neonatal care during the height of the pandemic. Analyses uncovered the idea that to keep safe meant to stay away, rendering a pattern of visits to their dying infant to perform care [38], in shifts. Change-overs became touch-points for parents to update one another on progress, and equally became points for confusion, something we believe is attributed to the pandemic circumstances and the associated restrictions. These points of misunderstanding or not receiving the correct information about the proposed or ongoing neonatal care, in a timely manner, echoes work undertaken in the wider field of maternity care studies during the current pandemic, whereby care – be it planning care or receiving it – was often not discussed or received in a way which could be easily understood or retained due to the increased reliance on virtual communication and the reduction in face-to-face provision of care [5, 32, 39].

As with maternal and neonatal care, restrictions on the care and support available to bereaved parents after a baby died were common, and included limiting attendees at funerals and religious ceremonies [40]. We found post-mortem and service investigations in the UK were not regularly explained face-to-face, but through video-calls, telephone, or in worst cases, by letter with no debriefing meeting. Parents also reported seeking support in new, virtual, ways (such as on-line counselling or support networks) which occasionally felt ineffective, especially when access to usual support networks and loved ones was not available. This often rendered bereavement care impersonal, which is again reminiscent of women’s entire journeys through pregnancy and childbirth from what we know from other research conducted during the pandemic [5, 6, 31, 32].

### Strengths, limitations, and future directions

A major strength of this study is the addition of ‘real-time’ knowledge about the experiences of maternity, neonatal, and bereavement care services in the UK during the COVID-19 global pandemic. This analysis provides the first insight into the implications of service reconfiguration for bereaved parents, and presents opportunities for shared learning for future health crises. It will be important for future research – as we attempt to understand the impact of the pandemic in different countries – to understand these differences agree on minimum care standards and levels of support in times of global health crises. One limitation was that only preliminary, descriptive, UK interview data could be presented, which may stilt the generalisability of our findings. However, the ongoing PUDDLES Global Collaboration will address this as more data become available from the other countries, with more in-depth analyses to follow. We would further add the lack of presentation of demographic data poses a limitation in the context of this study, and subsequent research should perhaps strive to find a workaround which would enable the presentation of demographic data, whilst ensuring absolute confidentiality and anonymity for participants who have the potential to be identified due to the nature of their relatively rare bereavement experiences. Ongoing research will benefit from increasing the number of interviews with parents who are bereaved through late miscarriage and neonatal death, and attempt to compare psychological responses between types of bereavement across the countries involved in PUDDLES. Future research should look in-detail at cross-cultural comparisons of bereavement and care through the COVID-19 pandemic, and potentially include perspectives from family members other than parents, and healthcare professionals to give a fuller picture of effects of service reconfiguration on pregnancy loss and perinatal death during the pandemic.

## Conclusion

The COVID-19 pandemic has created a second, uncontrollable rupture in bereaved parents’ lifecourses where they have had to navigate pregnancy loss or the death of their baby, whilst also navigating a global pandemic. We know from previous work that some pregnancy losses and perinatal deaths can be prevented with adequate care. However, during this pandemic, there has been a notable reduction of face-to-face care, with an increased reliance on virtual care, or the total removal of maternity care altogether [5, 6, 23], which could contribute to the rise in perinatal mortality [25]. Our findings, whilst preliminary, are important to document now, to help inform care and service provision as the pandemic continues and to recognise the impact of the removal of some aspects of maternity, neonatal, and/or bereavement care services. Our findings suggest blanket-changes to policies affecting maternity, neonatal, and bereavement care are not well-received and when a baby is thought to have or has indeed died, the presence of partners should be deemed essential to good quality care. Work should also be done by healthcare professionals to ensure care does not appear impersonal, which extends past clinical care to the provision of face-to-face post-mortem meetings, where it is safe to do so. Limiting birth partners to a woman’s partner and visitors to neonatal units was found to be acceptable in this study, as long as updates were documented for parents to pass on accurate information, however, this may not always be found to be acceptable, depending on the circumstances of the family. Likewise, limiting funeral attendees was permissible, as long as loved ones could spend time with the baby after birth. The COVID-19 pandemic has led to unprecedented levels of untimely death in the general population, however even after the pandemic subsides, some parents will continue to experience the premature death of their babies. With the information found through this collaboration and our analysis, we have evidenced ways to better support bereaved parents through care and grief.

## Supplementary Information

**Additional file 1.** The PUDDLES Study Interview Schedule: Late-Term Miscarriage.

**Additional file 2.** The PUDDLES Study Interview Schedule: Stillbirth.

**Additional file 3.** The PUDDLES Study Interview Schedule: Neonatal Death.

## Data Availability

The datasets generated and/or analysed during this study are not publicly available due to the sensitive nature of the interviews, but a de-identified dataset may be available from the corresponding author upon reasonable request.

## Abbreviations

COCOON: COntinuing care in COVID-19 Outbreak: a global survey of New, expectant and bereaved parent experiences
PUDDLES: The experiences of Parents who sUffer pregnancy loss and whose babies die During the panDemic: A quaLitative study of latE-term miscarriage, Stillbirth, and neonatal death
PPIE: Patient and Public Involvement and Engagement
Sands: Stillbirth and Neonatal Death Society
SARS-CoV-2: Severe Acute Respiratory Syndrome Coronavirus 2 (a.k.a. COVID-19)
Stillbirth CRE: The Centre of Research Excellence in Stillbirth, Australia
UK: United Kingdom
WHO: World Health Organization
