# Supplementary material for: Preliminary findings on the experiences of care for parents who suffered perinatal bereavement during the COVID-19 pandemic
Source: BMC Pregnancy Childbirth. 2021 Dec 22;21:840. doi: 10.1186/s12884-021-04292-5 (PMC8693591; doi:10.1186/s12884-021-04292-5)
Supplement: Supplementary file 1 — Additional file 1. The PUDDLES Study Interview Schedule: Late-Term Miscarriage. [file 12884_2021_4292_MOESM1_ESM.pdf]

# – The PUDDLES Study Interview Schedule: Late-Term Miscarriage –

The experiences of Parents who sUffer pregnancy loss and whose babies die During the panDemic: A quaLitative study of latE-term miscarriage, Stillbirth, and neonatal death.

Thank you for taking the time to be interviewed in relation to your recent experiences of Late-Term Miscarriage. May I start by saying how deeply sorry I am for your loss.

Introductions: You provided your details on the on-line COCOON survey and have agreed to talk to us in a little bit more depth about your experiences, and we are really grateful for you agreeing to do so. What I'd like to do in this interview is to ask you about your experiences of your Late-Term Miscarriage, in a bit more detail, and for you to tell me, in your own words, what happened including anything in relation to your experiences in the context of the COVID-19 pandemic. There are no right or wrong answers – we are interested in your experiences, perspectives, and opinions. We can take as long as you need to talk. We can pause or stop altogether at any time.

Do you have any questions before we begin? If not, I shall now start to record.

Opener:

People discuss miscarriage in different ways. I would like to use language that you feel comfortable with and so my first question is how do you refer to your experiences and whether you named your baby or not? If so, would you like me to use their name throughout the interview? [***N/B.*** Use <u>THEIR</u> language throughout].

In your own time, and in your own words, could you tell me about your experiences which led to you wanting to take part in this research?

[**N/B.** Only if no response, use: *Would you like to begin by telling me about your pregnancy?*] Probes (If unanswered by first monologue):

- What was your pregnancy like – any issues?
- What was your relationship with your HCP like?
- Was your pregnancy experience affected by COVID-19 lockdown restrictions?
- What was that experience like?
- Were you at home or in a healthcare setting?
- How did you feel? Physically? Emotionally?
- When did you realise you might have miscarried or when were you told that you had? Who told you?
- Could you tell me about these conversations – appropriate/sensitive?

How have communicated your miscarriage to friends, family, and other loved ones?

Probes (If unanswered by monologue):

- Who were the first people you told?
- How have people reacted to your loss?
- [If applicable] How have you communicated your miscarriage to your employer?

Have you felt people have understood your circumstances?

# – The PUDDLES Study Interview Schedule: Late-Term Miscarriage –

Bereavement & Grieving:

I'd now like to ask you about the time since your miscarriage until now.

Would you like to take a break, or are you okay to continue?

How have you been? Could you tell me about your thoughts and feelings throughout your experience of miscarriage?

Could you tell me about any support you have received in the time since your miscarriage?

This could be support from healthcare professionals, family, charities, or specialist bereavement/grief counselling services – please go into as much detail as you would like.

Probes (If unanswered by monologue):

- Who has provided the main source of support?
- What support has been the most helpful? Emotional? Practical?
- What were the things that helped? What didn't help?
- Do you think that the COVID-19 restrictions affect you accessing any services or support?
- How would you have liked to have been supported?

Advice Sharing:

My final questions are about your thoughts on care for Miscarriage more generally.

Would you like to take a break, or are you okay to continue?

When thinking about the care you received, what would you change, if anything?

Probes (If unanswered by monologue):

- What would be the most important change in your opinion?

Was there anything which was done particularly well during the course of your care?

Probes (If unanswered by monologue):

- What was the most important thing which helped you during this time?

How would you like to see Late-Term Miscarriage discussed and researched in the future?

And finally, do you have any advice for bereaved parents who have had the same or similar experiences to yourself?

I have come to the end of my interview questions, but is there anything at all that you would like to add that perhaps I haven't asked you about? Or do you have any questions for me?

If not, thank you very much for your time and again, my deepest condolences for your loss and should you require any further support, the resource list has links to charities and helplines which are still functioning during the pandemic.
