# Supplementary material for: Preliminary findings on the experiences of care for parents who suffered perinatal bereavement during the COVID-19 pandemic
Source: BMC Pregnancy Childbirth. 2021 Dec 22;21:840. doi: 10.1186/s12884-021-04292-5 (PMC8693591; doi:10.1186/s12884-021-04292-5)
Supplement: Supplementary file 2 — Additional file 2. The PUDDLES Study Interview Schedule: Stillbirth. [file 12884_2021_4292_MOESM2_ESM.pdf]

# – The PUDDLES Study Interview Schedule: Stillbirth –

The experiences of Parents who sUffer pregnancy loss and whose babies die During the panDemic: A quaLitative study of latE-term miscarriage, Stillbirth, and neonatal death.

Thank you for taking the time to be interviewed in relation to your recent experiences of Stillbirth. May I start by saying how deeply sorry I am for your stillbirth.

Introductions: You have already completed the on-line COCOON survey and have agreed to talk to us in a little bit more depth about your experiences, and we are really grateful for you agreeing to do so. What I'd like to do in this interview is to ask you about your experiences of your Stillbirth, in a bit more detail, and for you to tell me, in your own words, what happened including anything in relation to your experiences in the context of the COVID-19 pandemic. There are no right or wrong answers – we are interested in your experiences, perspectives, and opinions. We can take as long as you need to talk, and we can pause or stop altogether at any time.

Do you have any questions before we begin? If not, I shall now start to record.

Opener:

People discuss stillbirth in different ways. I would like to use language that you feel comfortable with and so my first question is how do you refer to your experiences and whether you named your baby or not? If so, would you like me to use their name throughout the interview? [***N/B.*** Use THEIR language throughout].

In your own time, and in your own words, could you tell me about your experiences which led to you wanting to take part in this research?

[**N/B.** Only if no response, use the following prompt: *Would you like to begin by telling me about your pregnancy?*]

Probes (If unanswered by first monologue):

- What was your pregnancy like – any issues?
- What was your relationship with your HCP like?
- Was your pregnancy or pregnancy experience affected by COVID-19 lockdown restrictions?
- When were you informed that your baby was likely to or had died? Who told you?
- Could you tell me about these conversations – appropriate/sensitive?
- Could you tell me about the time around labour and giving birth to your baby?
- Was your labour affected by COVID-19 lockdown restrictions? Did it have an effect on the decisions made about your labour? Did you understand your options?
- Who was allowed to be with you during labour? What support did you have around you? Were you able to return home before your labour to collect your belongings?

After their baby died:

I'd now like to ask you about what happened next.

Would you like to take a break, or are you okay to continue?

Could you tell me about what happened after your labour?

This might include any arrangements you made for registering your baby, your baby's funeral arrangements, and any postmortem investigations – please go into as much detail as you would like.

Probes (If unanswered by monologue):

- Have you had any follow-up appointments with your care provider?
- Do you think that the COVID-19 restrictions have affect the way you have been cared for after your stillbirth?
- Has it prevented you from accessing any appointments?

How have you communicated your baby has died to friends, family, and other loved ones?

Probes (If unanswered by monologue):

- Who were the first people you told?
- How have people reacted to your stillbirth?
- [If applicable] How have you communicated your stillbirth to your employer?

Have you felt people have understood your circumstances?

Bereavement & Grieving:

I'd now like to ask you about the time since your stillbirth until now.

Would you like to take a break, or are you okay to continue?

How have you been? Could you tell me a little about how you felt and what you were feeling during this time?

Could you tell me about any support you have received in the time since your stillbirth?

This could be support from healthcare professionals, family, charities, or specialist bereavement/grief counselling services – please go into as much detail as you would like.

Probes (If unanswered by monologue):

- Who has provided the main source of support?
- What support has been the most helpful? Emotional? Practical?
- What were the things that helped? What didn't help?
- Do you think that the COVID-19 restrictions affect you accessing any services or support?
- How would you have liked to have been supported?

Advice Sharing:

My final questions are about your thoughts on care for Stillbirth more generally.

Would you like to take a break, or are you okay to continue?

When thinking about the care you received, what would you change, if anything?

Probes (If unanswered by monologue):

- What would be the most important change in your opinion?

Was there anything which was done particularly well during the course of your care?

Probes (If unanswered by monologue):

- What was the most important thing which helped you during this time?

How would you like to see Stillbirth discussed and researched in the future?

And finally, do you have any advice for bereaved parents who have had the same or similar experiences to yourself?

I have come to the end of my interview questions, but is there anything at all that you would like to add that perhaps I haven't asked you about? Or do you have any questions for me?

If not, thank you very much for your time and again, my deepest condolences for the death of [your baby/baby's name] and should you require any further support, the resource list has links to charities and helplines which are still functioning during the pandemic.
